# Development and validation of a risk prediction model for motoric cognitive risk syndrome in older adults

**DOI:** 10.1007/s40520-024-02797-5

**Published:** 2024-07-13

**Authors:** Yaqin Li, Yuting Huang, Fangxin Wei, Tanjian Li, Yu Wang

**Affiliations:** 1 School of Nursing, Jinan University, Guangzhou, Guangdong Province China; 2 The Community Service Center of Jinan University, The First Affiliated Hospital of Jinan University, Tianhe District, Guangzhou, Guangzhou Province China

**Keywords:** Motoric cognitive risk syndrome, Older adults, Predictive model, Nomogram

## Abstract

**Objective:**

The objective of this study was to develop a risk prediction model for motoric cognitive risk syndrome (MCR) in older adults.

**Methods:**

Participants were selected from the 2015 China Health and Retirement Longitudinal Study database and randomly assigned to the training group and the validation group, with proportions of 70% and 30%, respectively. LASSO regression analysis was used to screen the predictors. Then, identified predictors were included in multivariate logistic regression analysis and used to construct model nomogram. The performance of the model was evaluated by area under the receiver operating characteristic (ROC) curve (AUC), calibration curves and decision curve analysis (DCA).

**Results:**

528 out of 3962 participants (13.3%) developed MCR. Multivariate logistic regression analysis showed that weakness, chronic pain, limb dysfunction score, visual acuity score and Five-Times-Sit-To-Stand test were predictors of MCR in older adults. Using these factors, a nomogram model was constructed. The AUC values for the training and validation sets of the predictive model were 0.735 (95% CI = 0.708–0.763) and 0.745 (95% CI = 0.705–0.785), respectively.

**Conclusion:**

The nomogram constructed in this study is a useful tool for assessing the risk of MCR in older adults, which can help clinicians identify individuals at high risk.

**Supplementary Information:**

The online version contains supplementary material available at 10.1007/s40520-024-02797-5.

## Background

The prevalence of dementia is increasing as the population ages. In fact, Chinese individuals with dementia make up approximately 25% of the global dementia population [1]. It is expected that by 2050, China will have nearly half of the global dementia population, which will place a heavy burden on the families of patients and the whole society. Due to the current lack of effective drug treatment methods for treating dementia [2], early diagnosis and timely implementation of preventive interventions are particularly important in addressing these challenges. Motor cognitive risk syndrome (MCR), a pre-dementia syndrome similar to mild cognitive impairment (MCI), was first proposed by Verghese et al. [3] in 2013. However, unlike MCI, the diagnosis of MCR does not require complex cognitive tests and neuroimaging, making it easier to carry out in clinical practice.

MCR is characterized by subjective cognitive complaints and decreased gait in elderly people without mobility disorders and dementia, which is a transitional state between normal cognition and dementia [3]. The worldwide prevalence of MCR has been reported to range from 2–27% [4, 5]. More and more evidence shows that MCR is an important predictor of dementia [6, 7] and older adults with MCR are at a higher risk of other adverse health outcomes such as falls, disabilities, and even mortality [8–10]. Therefore, it is crucial to concentrate on MCR and its modifiable risk factors to identify opportunities for early intervention in order to decrease the incidence of dementia.

At present, there is no report on the prediction model of MCR in the elderly population. This study aimed to identify and incorporate factors associated with MCR to construct a nomogram based on a model for predicting MCR in older adults.

## Materials and methods

### Study participants

The China Longitudinal Study of Health and Retirement (CHARLS) is a nationally representative longitudinal survey of Chinese people aged 45 years and older and their spouses [11]. We selected eligible participants from CHARLS 2015 for the analysis of this study. Once participants were identified, we used their IDs to obtain variables about their childhood experiences that were surveyed in 2014.

The inclusion criteria of this study were: (1) age ≥ 65 years; (2) complete cognitive complaints and gait speed information. Exclusion criteria were: (1) individuals with a history of dementia or disability.

### Assessment of motoric cognitive risk syndrome

MCR refers to the coexistence of subjective cognitive complaints and objective slow gait in individuals [12, 13]. In conjunction with data from the CHARLS database, cognitive complaints were evaluated using a self-reported question on memory: “How would you rate your memory at the present time?”. Participants with fair or poor reports were considered to have subjective cognitive complaints. Gait speed was measured by inviting participants to walk twice in a straight line of 2.5 m without a carpet at normal speed. We defined slow gait as ≥ 1.0 standard deviations below age- and sex- specific mean values of gait speed. Accordingly, cutoff values of slow gait for different age groups (65 to 69, 70 to 74, ≥ 75 years old) were 0.64, 0.60, 0.49 m/s for males and 0.58, 0.50, 0.39 m/s for females in 2015. Individuals presenting both cognitive complaints and slow gait were considered to have MCR.

### Predictors

#### Sociodemographic factors

Sociodemographic factors included age, sex, education level, marital status, permanent address, agricultural work experience, pension insurance, medical insurance, social participation, adverse childhood experiences, and childhood social isolation.

Jing-lin Yuan et al. [14] demonstrated that employment in agriculture was a risk factor for MCR. Furthermore, Donncha S. Mullin [15] conducted a longitudinal study and discovered that those who engaged in physical labour at an early age had a twofold increased risk of developing MCR in the future. In this study, the agricultural work experience was divided into two categories: yes and no.

According to the CHARLS questionnaire, social participation can be divided into four categories: social entertainment type, voluntary public welfare type, economic activity type and labor participation type. If the participant did not engage in any of the above types of social participation, it was recorded as 0, and if there was one type of social participation, it was recorded as 1. Participants’ social participation was categorized into four levels: none, poor, fair, and good.

In 2014, the CHARLS database investigated the life history of the participants. A longitudinal investigation conducted by Haixu Liang [16] revealed that individuals who had experienced three or more adverse childhood experiences (ACEs) exhibited a heightened risk of MCR compared to those who had not. Based on prior research [17, 18], we incorporated 10 indicators of ACEs. Each adversity was coded as either present (“1”) or absent (“0”). We calculated the total number of ACEs encountered and divided them into 3 groups (0, 1, and ≥ 2 ACEs) based on the total number.

The current research has indicated that individuals who had experienced a greater degree of social isolation during their childhood were more likely to exhibit impaired cognitive and behavioural abilities in later life [19]. In this study, the definition of childhood social isolation variable follows the conception proposed by Caspi et al. [20], encompassing social exclusion and withdrawal. Four questions were selected from the 2014 CHARLS Life History Survey. Responses of “rarely or never” were coded as 0, “sometimes” as 1, and “often” as 2. Subsequently, the scores for each question were summed.

#### Behavioral factors

Behavioral factors comprised drinking, smoking, afternoon nap, sleep quality, and nighttime sleep. Sleep quality was assessed based on the “my sleep is restless” response and divided into four groups based on how often this statement occurred during the week. Afternoon nap and nighttime sleep were classified based on the duration reported by participants in the past month.

#### Physical health factor

Based on previous research and our expertise [21–25], potential predictors of MCR included multiple morbidity, arthritis or rheumatism, hypertension, chronic pain, fall, hospitalization, activity of daily living (ADL) damaged, limb dysfunction score, visual acuity score, self-perceived health status, hearing, malnutrition and appendicular skeletal muscle mass (AMS).

Multiple morbidity was defined as having two or more chronic conditions. ADL damaged was assessed using 11 questions in the CHARLS questionnaire, covering activities such as dressing, bathing, eating, cooking, and so on. Participants who had difficulty with any of these activities and needed assistance were considered to have “ADL damaged”.

The limb dysfunction score assesses physical functional limitations, with participants rating the difficulty level in tasks such as running or jogging 1 km, walking 1 km, walking 100 m, carrying weights over 10 jin, and so on. Participants received 4 points if they were unable to complete a task, and 1 point if no difficulty arose. Add up the scores from 9 questions.

Visual acuity in CHARLS data included the evaluation of near and far vision, with scores ranging from 1 for very good to 4 for poor. The average of the near and far visual acuity scores was taken to determine the participants’ overall visual acuity score.

We used the definition of malnutrition from the European Society for Parenteral and Enteral Nutrition and Metabolism (ESPEN) [26]. Participants were classified as malnourished if they met any of the following criteria: (1) BMI < 18.5 kg/m^2^; (2) More than 10% weight loss over an unspecified period; (3) Participants under 70 years old with a BMI < 20 kg/m2 and those aged 70 and above with a BMI < 22 kg/m^2^.

Muscle mass was estimated using an anthropometric equation validated in Chinese individuals [27]: ASM = 0.193×weight (kg)+0.107×height (cm)-4.157×sex -0.037×age (years)-2.631. In this equation, sex is assigned as 1 for males and 2 for females.

#### Mental health factors

Mental health factors included depression and loneliness. Depression was evaluated using the Centers for Epidemiology Studies Depression Scale (CES-D) [28]. In this scale, a total score of ≤ 10 indicated the absence of depressive symptoms, 10–19 suggested mild depressive symptoms, and ≥ 20 indicated severe depressive symptoms. Loneliness was assessed by the question, “Did you feel lonely last week?”.

#### Physical measurement index

Physical measures comprised Body Mass Index (BMI), waist circumference, systolic and diastolic blood pressure, grip strength, weakness, the Five-Times-Sit-To-Stand (FTSS) test, and blood test results. According to the 2019 Consensus of the Asian Sarcopenia Working Group [29], weakness is defined as male grip strength < 28 kg and female grip strength < 18 kg. During the FTSS test, participants were instructed to stand up straight, and then sit in a standard-height armless chair five times consecutively, with a stopwatch recording the entire process [30].

The specific contents of the above predictors are shown in Table 1 of the additional file.

### Statistical analysis

Statistical analysis was conducted with R software and SPSS Statistics 26.0. Two-tailed were used in all tests, and significance level was *P* < 0.05. Categorical variables were presented as frequencies and percentages, while continuous variables were reported as mean ± standard deviation (SD) or median with interquartile range (IQR). Group comparisons were conducted using t-tests, chi-square tests, and nonparametric tests as appropriate. The dataset was randomly split into a training set (*n* = 2773) and a validation set (*n* = 1189) in a 7:3 ratio [31].

First, the least absolute shrink age and selection operator (LASSO) regression analysis was conducted on the training dataset [32] to identify predictors of MCR. Ten-fold cross-validation was utilized to identify the optimal tuning parameters (λ) for the LASSO regression, and important features were selected. Subsequently, the predictive factors were incorporated into the multivariate logistic regression analysis. Lastly, predictive factors with p-value < 0.05 were used to develop the nomogram. The maximum missing value of all extracted variables does not exceed 20%, and multiple imputation was used to handle missing data [33].

The area under the receiver operating characteristic (ROC) curve (AUC) was used to assess the discrimination ability of the model. Calibration curves and the Hosmer-Lemeshow goodness-of-fit test were employed to evaluate the agreement between predicted and observed values in the nomogram. Furthermore, clinical validity of the predictive nomogram was assessed through decision curve analysis (DCA).

## Results

### Participant characteristics

The study included 3962 elderly participants, comprising 1964 males (49.6%) and 1998 females (50.4%). Table 1 presents the demographic and clinical characteristics of the participants. The prevalence of MCR was 13.3% (528/3962). A comparison of the distribution of variables between the training and validation sets is provided in Table 3 of the supplementary file, showing small differences between the two groups.

**Table 1:** Baseline characteristics of the study population

Variables	Total	Non-MCR	MCR	*P*
	3962	*n* = 3434	*n* = 528	
AMS	16.09 (4.25)	16.19 (4.22)	15.41 (4.36)	<0.001
Grip strength (kg)	25.12 (8.54)	25.65 (8.39)	21.72 (8.74)	<0.001
Limb dysfunction score	14.10 (5.20)	13.55 (8.39)	17.65 (6.38)	<0.001
Visual acuity score	2.82 (0.82)	2.79 (0.83)	3.07 (0.70)	<0.001
Childhood social isolation	1.10 (1.33)	1.07 (1.31)	1.29 (1.44)	0.001
BMI	23.14 (4.11)	23.14 (3.92)	23.14 (5.18)	0.985
Waist	84.55 (13.84)	84.63 (13.72)	84.03 (14.55)	0.354
FTSS	10.87 (13.84)	10.45 (4.00)	13.67 (6.14)	<0.001
Bl-hdl	51.72 (12.41)	51.68 (12.45)	52.01 (12.14)	0.567
Bl-cysc	0.95 (0.26)	0.95 (0.24)	0.99 (0.33)	0.002
Bl-crp	3.11 (6.79)	2.94 (6.33)	4.27 (9.14)	0.001
Bl-glu	104.10 (32.52)	103.78 (31.09)	106.23 (40.57)	0.185
Bl-cho	184.11 (35.82)	184.61 (35.52)	180.85 (37.56)	0.025
Bl-bun	16.48 (4.93)	16.45 (4.92)	16.65 (4.99)	0.396
Bl-ua	5.07 (1.43)	5.09 (1.44)	4.96 (1.42)	0.054
Bl-wbc	5.97 (1.81)	5.95 (1.76)	6.12 (2.11)	0.040
Bl-hgb	13.44 (1.84)	13.46 (1.84)	13.31 (1.86)	0.091
Bl-hct	40.91 (5.59)	40.96 (5.57)	40.62 (5.69)	0.198
Bl-crea	0.85 (0.31)	0.85(0.31)	0.87 (0.33)	0.276
Bl-ldl	103.51 (28.84)	103.92 (28.41)	100.89 (31.42)	0.025
Bl-hbalc	6.08 (0.98)	6.06 (0.93)	6.15 (1.29)	0.119
Systolic pressure	134.21 (21.13)	134.06 (20.84)	135.20 (22.92)	0.283
Diastolic pressure	73.98 (10.97)	74.00 (11.01)	73.81 (10.72)	0.711
Arthritis or rheumatism(%)	1569 (39.6)	1339 (39)	230 (43.6)	0.046
Hypertension(%)	1197 (30.2)	1029 (30)	168 (31.8)	0.388
Malnutrition(%)	1390 (35.1)	1186 (34.5)	204 (38.6)	0.066
Gender (%)				0.097
Male	1964 (49.6)	1720 (50.1)	244 (46.2)	
Female	1998 (50.4)	1714 (49.9)	284 (53.8)	
Weakness (%)	1421 (35.9)	1136 (33.1)	285 (54)	<0.001
Age, years (%)				0.497
<75	1750 (44.2)	1524 (44.4)	226 (42.8)	
≥ 75	2212 (55.8)	1910 (55.6)	302 (57.2)	
Marital status(%)				<0.001
Married	2952 (74.5)	2592 (75.5)	360 (68.2)	
Unmarried	1010 (25.5)	842 (24.5)	168 (31.8)	
Educational level(%)				<0.001
Primary	3257 (82.2)	2783 (81)	474 (89.8)	
Secondary	636 (16.1)	583 (17)	53 (10)	
Tertiary	69 (1.7)	68 (2)	1 (0.2)	
Permanent address (%)				0.028
Urban	993 (25.1)	881 (25.7)	112 (21.2)	
Rural	2969 (74.9)	2553 (74.3)	416 (78.8)	
Agricultural work experience(%)				0.090
No	1452 (36.6)	1241 (36.1)	211 (40)	
Yes	2510 (63.4)	2193 (63.9)	317 (60)	
Currently smoking (%)	1057 (26.7)	917 (26.7)	140 (26.5)	0.927
Currently drinking (%)	1259 (31.8)	1136 (33.1)	123 (23.3)	<0.001
Nighttime sleep duration(%)				0.002
<6 h	1431 (36.1)	1199 (34.9)	232 (43.9)	
6–8 h	2072 (52.3)	1840 (53.6)	232 (43.9)	
≥ 9 h	459 (11.6)	395 (11.5)	64 (12.1)	
Afternoon nap(%)				0.769
0 h	1595 (40.2)	1380 (40.2)	215 (40.7)	
<1 h	648 (16.4)	572 (16.7)	76 (14.4)	
≥ 1 h	1719 (43.4)	1482 (43.2)	237 (44.9)	
Sleep quality (%)				<0.001
Rarely or none of the time	2031 (51.3)	1808 (52.6)	223 (42.2)	
Some or a little of the time	548 (13.8)	475 (13.8)	73 (13.8)	
Occasionally or a moderate amount of the time	513 (12.9)	419 (12.2)	94 (17.8)	
Most or all of the time	870 (22)	732 (21.3)	138 (26.1)	
Multiple morbidity (%)				0.008
No	2127 (53.7)	1872 (54.5)	255 (48.3)	
Yes	1835 (46.3)	1562 (45.5)	273 (51.7)	
Chonic pain (%)	1257 (31.7)	1005 (29.3)	252 (47.7)	<0.001
Fall (%)				0.004
No	3205 (80.9)	2802 (81.6)	403 (76.3)	
Yes	757 (19.1)	632 (18.4)	125 (23.7)	
ADL damaged (%)				<0.001
No	3172 (80.1)	2853 (83.1)	319 (60.4)	
Yes	790 (19.9)	581 (16.9)	209 (39.6)	
Self-perceived health status (%)				<0.001
Good	2833 (71.5)	2543 (74.1)	290 (54.9)	
Poor	1129 (28.5)	891 (25.9)	238 (45.1)	
Hearing (%)				<0.001
Good	1084 (27.4)	988 (28.8)	96 (18.2)	
Poor	2878 (72.6)	2446 (71.2)	432 (81.8)	
Dpression (%)				<0.001
No depressive symptoms	2817 (71.1)	2510 (73.1)	307 (58.1)	
Mild depressive symptoms	873 (22)	711 (20.7)	162 (30.7)	
Severe depressive symptoms	272 (6.9)	213 (6.2)	59 (11.2)	
Aloneness (%)				<0.001
No	2848 (71.9)	2507 (73)	341 (64.6)	
Yes	1114 (28.1)	927 (27)	187 (35.4)	
Social participation (%)				<0.001
None	800 (20.2)	641 (18.7)	159 (30.1)	
Poor	1746 (44.1)	1518 (44.2)	228 (43.2)	
Fair	1072 (27.1)	954 (27.8)	118 (22.3)	
Good	344 (8.7)	321 (9.3)	23 (4.4)	
Hospitalization history (%)	699 (17.6)	574 (16.7)	125 (23.7)	<0.001
Endowment insurance (%)				0.102
No	1149 (29)	980 (28.5)	169 (32)	
Yes	2813 (71)	2454 (71.5)	359 (68)	
Medical insurance (%)				0.461
No	414 (10.4)	354 (10.3)	60 (11.4)	
Yes	3548 (89.6)	3080 (89.7)	468 (88.6)	
Adverse childhood experience (%)				0.173
0	82 (2.1)	67 (2)	15 (2.8)	
1	698 (17.6)	598 (17.4)	100 (18.9)	
≥ 2	3182 (80.3)	2769 (80.6)	413 (78.2)	

AMS, appendicular skeletal muscle mass; BMI, Body Mass Index; FTSS, Five-Times-Sit-To-Stand; Bl-hdl, high density lipoprotein cholesterol; Bl-cysc, cystatin c; Bl-crp, c-reactive protein; Bl-glu, glucose; Bl-cho, total cholesterol; Bl-bun, blood urea nitrogen; Bl-ua, uric acid; Bl-wbc, white blood cell; Bl-hgb, hemoglobin; Bl-hct, hematocrit; Bl-crea, creatinine; Bl-ldl, low density lipoprotein cholesterol; Bl-hbalc, glycated hemoglobin

### Predictive model development

**Fig. 1:**
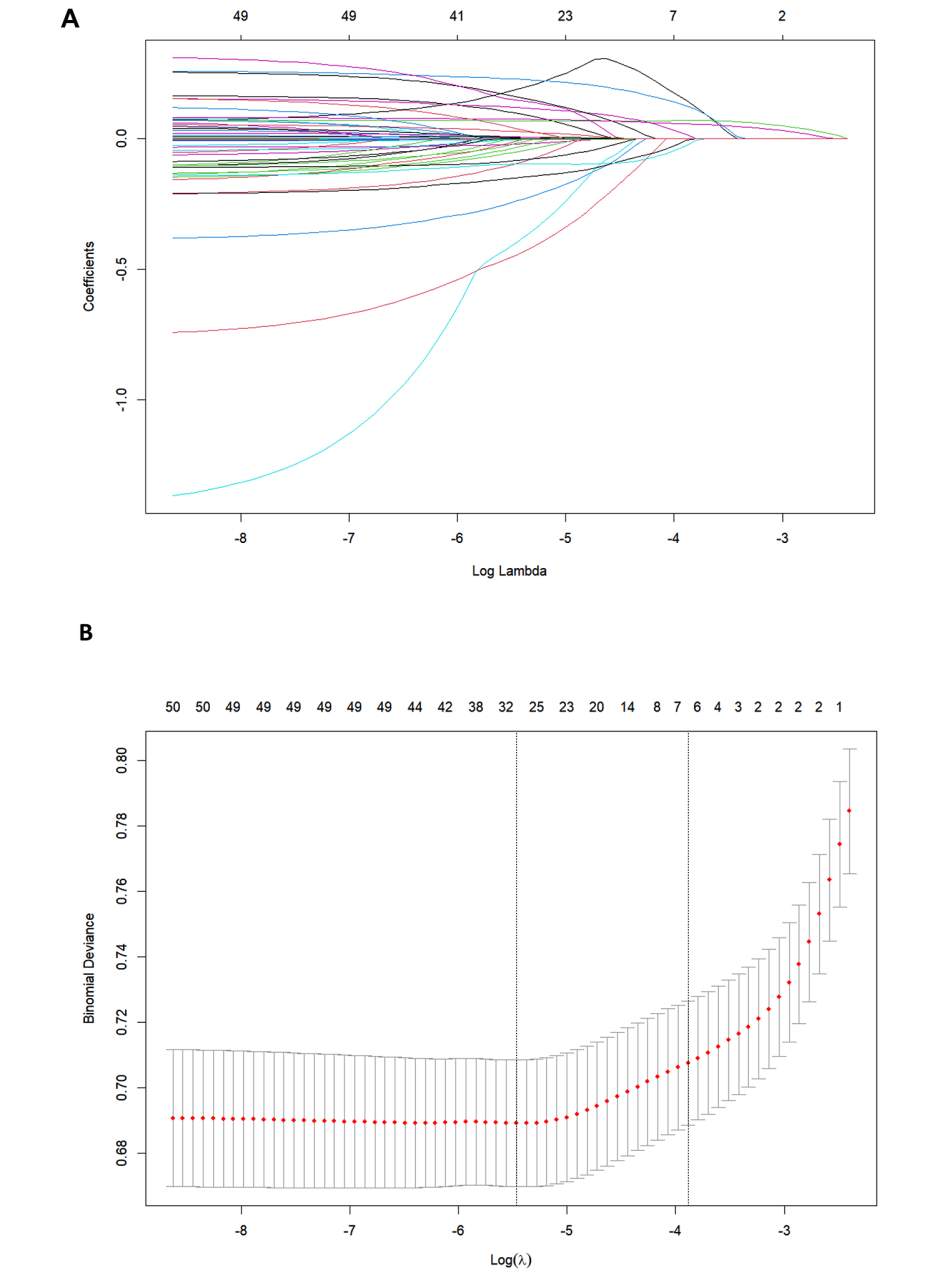
Demographic and clinical feature selection using the LASSO regression model (**A**). According to the logarithmic (lambda) sequence, a coefcient profle was generated, and non-zero coefcients were produced by the optimal lambda (**B**). The optimal parameter (lambda) in the LASSO model was selected via tenfold cross-validation using minimum criteria

**Fig. 2:**
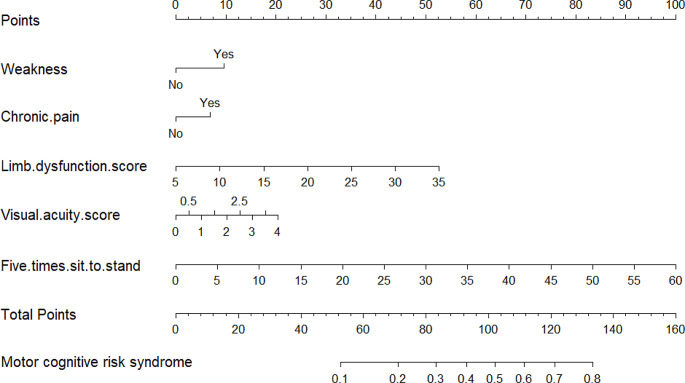
Nomogram

Potential predictors of MCR were identified based on Lasso regression analysis (Fig. 1A and B). These potential factors were integrated into the logistic regression model, and the results are presented in Table 2. A variance inflation factor (VIF) test was conducted, with VIF values for all variables being < 4. Then, we proceeded to develop the nomogram (Fig. 2). Use the appropriate scale of the nomogram to calculate an individual score for each risk factor and subsequently aggregate the scores. By comparing the corresponding percentages at the bottom, the predicted risk of MCR in older adults can be determined.

**Table 2:** The prediction model with multivariate logistic regression

Variable	Multivariate analysis OR (95%CI)	*P*-value
Weakness	1.53 (1.20–1.95)	<0.001
Chronic pain	1.36 (1.05–1.75)	<0.05
Limb dysfunction score	1.08 (1.06–1.11)	<0.001
Visual acuity score	1.25 (1.07–1.47)	<0.01
FTSS	1.08 (1.05–1.10)	<0.001

*OR*,odds ratio; *CI*,confdence interval; FTSS, Five-Times-Sit-To-Stand

### Predictive model validation

#### Discrimination

**Fig. 3:**
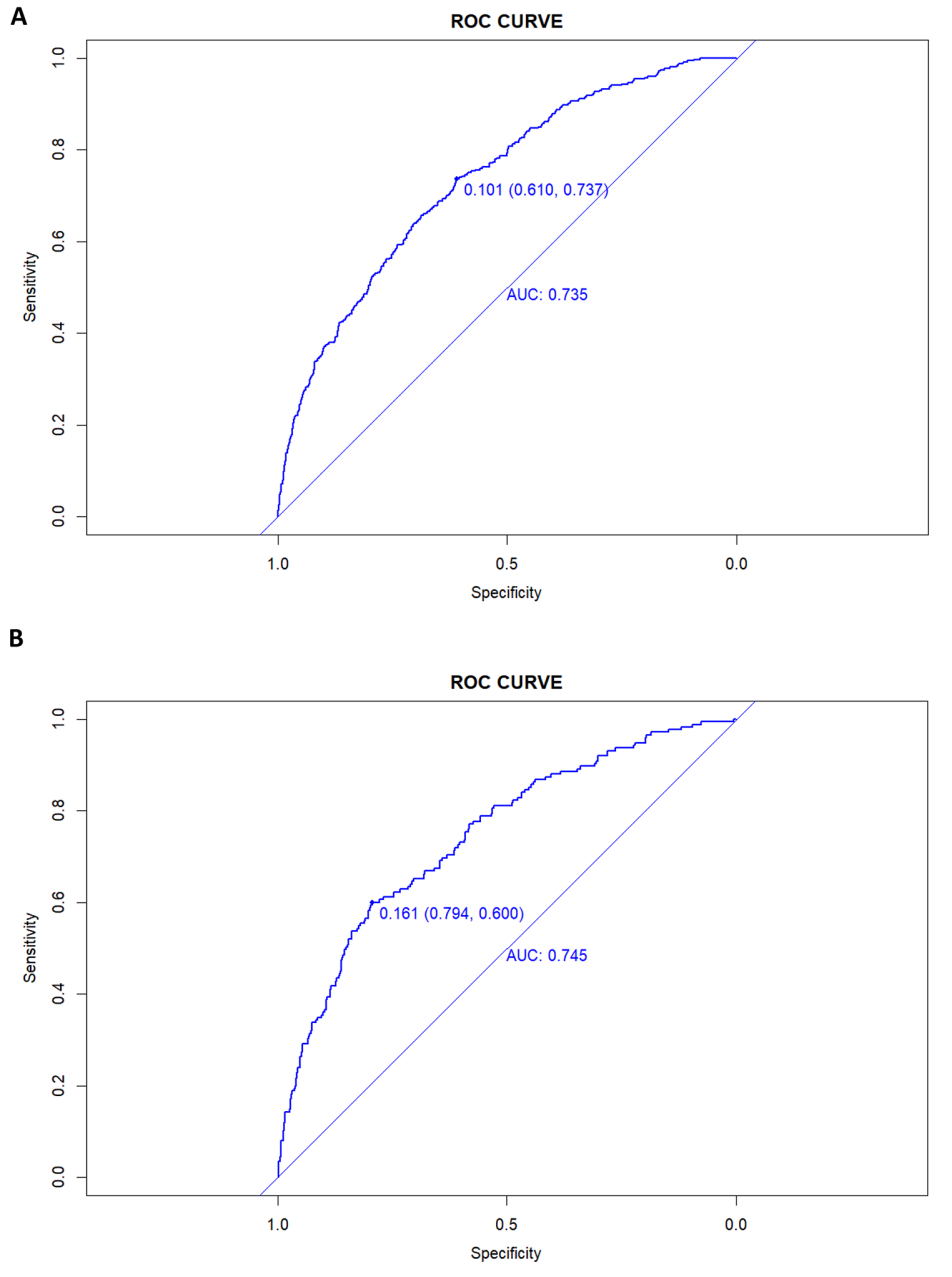
A nomogram ROC curve generated from the training dataset (**A**). A nomogram ROC curve generated using the validation dataset (**B**)

As shown in Fig. 3A and B, the prediction model had an AUC value of 0.735 (95%CI = 0.708–0.763), specificity of 0.610, and sensitivity of 0.737 in the training set. The AUC value in the validation set was 0.745 (95%CI = 0.705–0.785), with a specificity of 0.794 and a sensitivity of 0.600.

#### Calibration of the predictive model

**Fig. 4:**
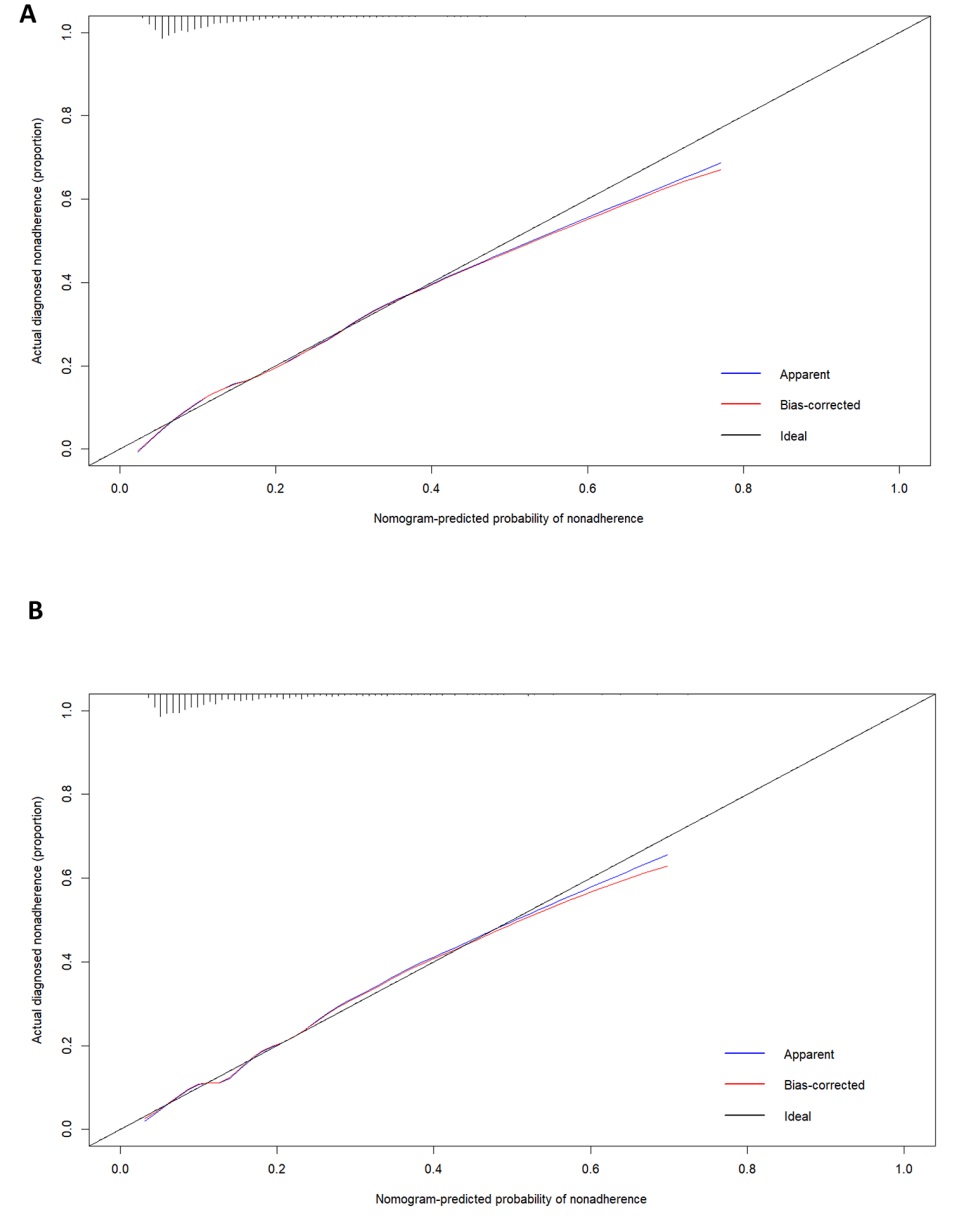
Calibration plots for the training dataset (**A**) and the validation dataset (**B**)

The Hosmer-Lemeshow goodness-of-fit test indicated excellent fit of the model for both the training (χ2 = 9.3378, *p* = 0.4067) and validation (χ2 = 12.2162, *p* = 0.2014) sets. Calibration plots for both the training and validation sets are displayed in Fig. 4A and B, demonstrating high consistency between the predicted and actual probabilities of MCR.

#### Evaluation of clinical validity

**Fig. 5:**
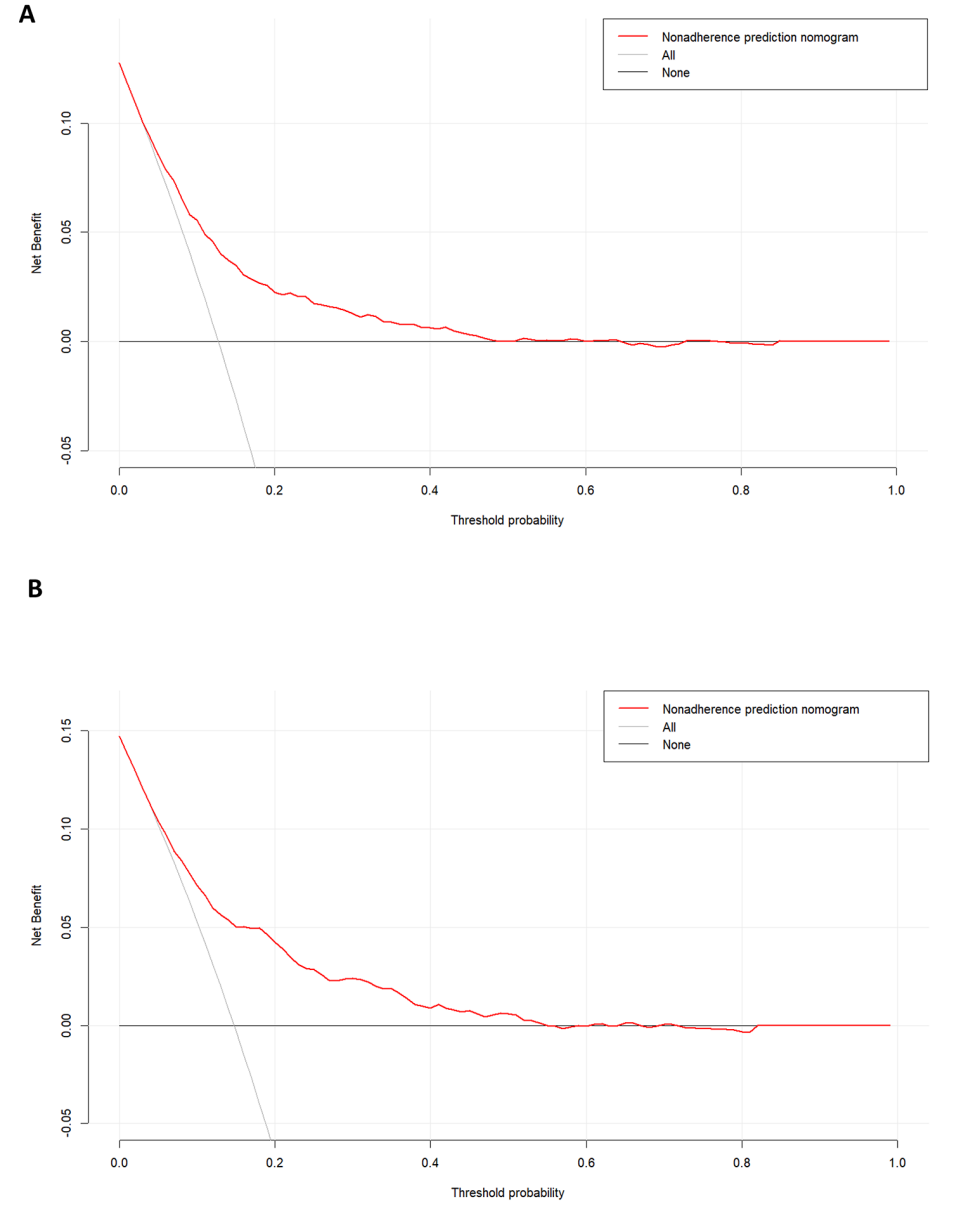
DCA curve for the training dataset (**A**). DCA curve for the validation dataset (**B**)

DCA has two reference lines, one reflecting the net benefit of not receiving any treatment, and the other reflecting the net benefit of all patients receiving treatment. The results showed that both the training group (Fig. 5A) and the validation group (Fig. 5B) achieved greater net benefits when using this predictive model for clinical decision-making compared to not treating or treating all patients, indicating that the nomogram model had superior net benefit and predictive accuracy.

## Discussion

In our study, the prevalence of MCR was 13.3%, which is slightly different from the 6.5% prevalence of MCR among rural elderly people in Guizhou surveyed by scholar Jiang Yun [34] and the 9.6% prevalence of MCR among elderly people in Beijing surveyed by Chhetri et al [35]. This suggests that more research is needed in the future to explore the reasons for the differences in prevalence in different regions. Prior studies have indicated that MCR, as a novel concept for identifying dementia risk, enhances our comprehension of dementia’s pathophysiological mechanisms and enables early dementia prevention [36]. We utilized the LASSO method for features selection, eventually developing and validating the model using five crucial features, and created the nomogram.

The study revealed that weakness and prolonged Five-Times-Sit-To-Stand (FTSS) test duration were independent predictors of MCR. Weakness in this study meant low grip strength. Our finding aligns with prior researches, indicating a correlation between decreased grip strength and a higher prevalence of MCR [37]. For example, Dian Jiang et al. [38] identified a notable linear dose-response association between grip strength and MCR, with the probability of MCR rising by 3% for each 1-kilogram decrease in hand grip strength (HGS). Studies have shown various potential pathophysiological mechanisms linking reduced grip strength to cognitive decline, such as the generation of pro-inflammatory cytokines [39] and elevated white matter intensity [40]. In addition, higher grip strength may reflect regular physical activity, which has been associated with a reduced risk of dementia in some epidemiological or clinical studies [41]. Regular exercise can help maintain greater muscle strength and enhance cardiovascular function after middle age, contributing to improved health in later years and cognitive function. Similarly, as a validated measure of muscle strength, our study revealed a positive correlation between prolonged FTSS duration and an increased risk of MCR, aligning with the outcomes of Han Xiao’s research [42]. The FTSS test examines participants’ ability to transition from sitting to standing positions, a process that is influenced by balance and multiple sensorimotor factors [43], which may explain why FTSSis associated with cognitive function. The results of our study implied that improving lifestyle and exercise may be ways to prevent MCR syndrome, dementia or other adverse consequences.

Our predictive model showed that chronic pain was also associated with MCR. This correlation has been supported by prior research. Liang Haixu et al. [44] tracked 3711 elderly individuals over a 4-year period and observed that those with chronic pain were about 1.5 times more prone to MCR compared to those without chronic pain (HR 1.5, 95% CI 1.23–1.99). Actually, pain is closely related to cognition, as both can trigger similar brain pathways involving structures like the amygdala, hippocampus, and nucleus accumbens [45]. The link between pain and cognitive decline may be attributed to the atrophy of overlapping neural networks. Wenhui Zhao et al. [46] found that compared with pain-free individuals, individuals with multisite chronic pain were associated with significantly higher dementia risk, broader and faster cognitive impairment, and greater hippocampal atrophy. In addition, a prominent theory has established a connection between pain and cognitive function. The pain interruption model posits that pain impairs cognitive function through distraction, a hypothesis corroborated by clinical research [47]. Our study highlighted the importance of focusing on the elderly population with chronic pain in the future, emphasizing the necessity of making safer and more effective treatment choices for this group.

The study also found that a high limb dysfunction score was an independent predictor of MCR. At present, more and more evidence suggests the significance of limb function in identifying cognitive decline [48–50]. Individuals with cognitive impairment may experience overloading of their brain networks when facing complex tasks involving cognitive responses and functional mobility, which manifests as limb dysfunction [51, 52]. Moreover, people with poor limb function usually have reduced venous return, which may lead to vertebral hypoperfusion and a decrease in cognitive function [53, 54]. Therefore, incorporating limb dysfunction score into routine assessments of older adults could help healthcare providers stratify risk to develop interventions for older adults at high risk of MCR.

Moreover, this study found that reduced visual function was associated with a higher risk of MCR. Current studies agree that the underlying mechanism between visual impairment and cognitive decline is unclear. Only a few hypotheses have been proposed for this association. The sensory loss impact theory suggests that vision loss prevents older adults from engaging in activities that are essential to maintaining cognitive function [55]. Another hypothesis is the co-cause hypothesis, which proposes that visual and cognitive decline is due to common factors like inflammation and central nervous system dysfunction [56, 57]. Vision assessment is cost-effective and non-invasive. Timely assessment of vision in older adults, followed by appropriate interventions, could offer additional benefits. For example, Haotian Lin [58] conducted a controlled clinical trial and found that the improvement of vision after cataract surgery was related to the improvement of cognitive ability and the increase of cortical gray matter volume.

The study is based on a large and nationally representative survey conducted in China, which enhances the generalizability and reliability of our findings. In addition, this study is the first attempt to establish a MCR risk prediction model. Our predictive model can help healthcare professionals screen older adults at high risk of MCR in order to develop early prevention and intervention measures. However, our research also has some limitations. Firstly, it should be noted that our study was based on retrospective cross-sectional data, which could not determine the presence of risk factors before the occurrence of MCR. Future studies could incorporate data from follow-up patients to enhance the current predictive model and provide more reliable insights into the occurrence of MCR in older adults in the community. Secondly, some variables in CHARLS were evaluated through self-report, which may be influenced by recall bias. Additionally, our model was developed and validated based on population data from China, and the research results may not be applicable to other races.

## Conclusions

This study developed and validated a nomogram model for predicting MCR occurrence in older adults. Our nomogram model combined weakness, FTSS, chronic pain, limb dysfunction score and visual acuity score, and has been internally validated as a useful risk assessment tool. This prediction model will be of great value for screening MCR in the elderly.

### Electronic supplementary material

Below is the link to the electronic supplementary material.

Supplementary Material 1

## Data Availability

The data are publicly available on the China Health and Retirement Longitudinal Study website. https://charls.charlsdata.com/pages/data/111/zh-cn.html.

## References

[1] Jia L, Quan M, Fu Y (2020). Dementia in China: epidemiology, clinical management, and research advances. Lancet Neurol.

[2] Castro CB, Costa LM, Dias CB (2023). Multi-domain Interventions for Dementia Prevention - A systematic review. J Nutr Health Aging.

[3] Verghese J, Annweiler C, Ayers E (2014). Motoric cognitive risk syndrome: multicountry prevalence and dementia risk. Neurology.

[4] Meiner Z, Ayers E, Verghese J (2020). Motoric cognitive risk syndrome: a risk factor for cognitive impairment and dementia in different populations. Annals Geriatric Med Res.

[5] ZhiFei W, SiHan P, JiaLin W (2022). Prevalence of motoric cognitive risk syndrome among older adults: a systematic review and meta-analysis. Aging Ment Health.

[6] Mullin DS, Cockburn A, Welstead M (2022). Mechanisms of motoric cognitive risk-hypotheses based on a systematic review and meta-analysis of longitudinal cohort studies of older adults. Alzheimers Dement.

[7] Verghese J, Wang C, Bennett DA (2019). Motoric cognitive risk syndrome and predictors of transition to dementia: a multicenter study. Alzheimer’s Dement J Alzheimer’s Assoc.

[8] WeiWei L, BangZhong L, MinZhi L (2023). Association between motoric cognitive risk syndrome and future falls among Chinese community-dwelling elderly: a nationwide cohort study. Brain Behav.

[9] Anying B, Weimin B, Hepeng J (2022). Motoric cognitive risk syndrome as a predictor of incident disability: a 7 year follow-up study. Front Aging Neurosci.

[10] Beauchet O, Sekhon H, Launay CP (2019). Motoric cognitive risk syndrome and mortality: results from the EPIDOS cohort. Eur J Neurol.

[11] Yaohui Z, Yisong H (2014). Cohort profile: the China Health and Retirement Longitudinal Study (CHARLS). Int J Epidemiol.

[12] Liang H, Fang Y (2023). Longitudinal association between falls and motoric cognitive risk syndrome among community-dwelling older adults. Geriatr Nurs.

[13] Liang H, Fang Y (2023). Association of polypharmacy and motoric cognitive risk syndrome in older adults: a 4-year longitudinal study in China. Arch Gerontol Geriatr.

[14] Yuan JL, Zhao RX, Ma YJ (2021). Prevalence/potential risk factors for motoric cognitive risk and its relationship to falls in elderly Chinese people: a cross-sectional study. Eur J Neurol.

[15] Mullin DS, Stirland LE, Russ TC (2023). Socioeconomic status as a risk factor for motoric cognitive risk syndrome in a community-dwelling population: a longitudinal observational study. Eur J Neurol.

[16] Liang H, Fang Y (2023). Associations between adverse childhood experiences and motoric cognitive risk syndrome: a prospective, longitudinal, observational, cohort study. Int J Geriatr Psychiatry.

[17] Li L, Haoxiang WH, Ciyong L (2021). Adverse childhood experiences and subsequent chronic diseases among Middle-aged or older adults in China and associations with demographic and socioeconomic characteristics. JAMA Netw open.

[18] Lin L, Cao B, Chen W (2022). Association of Adverse Childhood Experiences and social isolation with later-life cognitive function among adults in China. JAMA Netw Open.

[19] Wang G, Cheng Z, Zhou Y (2023). The effect of childhood social isolation on behavioral cognition in Chinese middle-aged and older adults: the moderating effect of family support. Arch Gerontol Geriatr.

[20] Caspi A, Harrington H, Moffitt TE (2006). Socially isolated children 20 years later: risk of cardiovascular disease. Arch Pediatr Adolesc Med.

[21] Feiyang X, Yizhong W, Jun Z (2023). Association of multimorbidity patterns with motoric cognitive risk syndrome among older adults: evidence from a China longitudinal study. Int J Geriatr Psychiatry.

[22] Sun X, Harris KE, Hou L (2022). The prevalence and associated factors of motoric cognitive risk syndrome in multiple ethnic middle-aged to older adults in west China: a cross-sectional study. Eur J Neurol.

[23] Lau H, Ludin AFM, Shahar S (2019). Factors associated with motoric cognitive risk syndrome among low-income older adults in Malaysia. BMC Public Health.

[24] Du H, Yu M, Xue H (2022). Association between Sarcopenia and cognitive function in older Chinese adults: evidence from the China health and retirement longitudinal study. Front Public Health.

[25] White SA, Ward N, Verghese J (2020). NUTRITIONAL RISK STATUS, DIETARY INTAKE AND COGNITIVE PERFORMANCE IN OLDER ADULTS WITH MOTORIC COGNITIVE RISK SYNDROME. JAR Life.

[26] Cederholm T, Bosaeus I, Barazzoni R (2015). Diagnostic criteria for malnutrition–An ESPEN Consensus Statement. Clin Nutr.

[27] Wen X, Wang M, Jiang CM (2011). Anthropometric equation for estimation of appendicular skeletal muscle mass in Chinese adults. Asia Pac J Clin Nutr.

[28] Mohebbi M, Van Nguyen, McNeil JJ (2018). Psychometric properties of a short form of the Center for epidemiologic studies Depression (CES-D-10) scale for screening depressive symptoms in healthy community dwelling older adults. Gen Hosp Psychiatry.

[29] Chen LK, Woo J, Assantachai P (2020). Asian Working Group for Sarcopenia: 2019 Consensus Update on Sarcopenia diagnosis and treatment. J Am Med Dir Assoc.

[30] Sekhon H, Launay CP, Chabot J (2018). Motoric cognitive risk syndrome: could it be defined through increased five-Times-Sit-to-stand Test Time, rather than slow walking speed?. Front Aging Neurosci.

[31] WenTao W, YuanJie L, AoZi F (2021). Data mining in clinical big data: the frequently used databases, steps, and methodological models. Military Med Res.

[32] JiaYu H, Ying W, XiangMin T (2021). When to consider logistic LASSO regression in multivariate analysis?. Eur J Surg Oncology: J Eur Soc Surg Oncol Br Association Surg Oncol.

[33] Morris TP, White IR, Royston P (2014). Tuning multiple imputation by predictive mean matching and local residual draws. BMC Med Res Methodol.

[34] Yun J, Quan-xiang Z, Xing Y (2023). Analysis on prevalence and influencing factors of motoric cognitive risk syndrome in rural Guizhou Elderly. Mod Prev Med.

[35] Chhetri JK, Han C, Dan X (2020). Motoric cognitive risk syndrome in a Chinese older Adult Population: Prevalence and Associated factors. J Am Med Dir Assoc.

[36] Semba RD, Tian Q, Carlson MC (2020). Motoric cognitive risk syndrome: integration of two early harbingers of dementia in older adults. Ageing Res Rev.

[37] Jia S, Zhao W, Ge M (2023). Association of Handgrip Strength Weakness and asymmetry with incidence of motoric cognitive risk syndrome in the China Health and Retirement Longitudinal Study. Neurology.

[38] Dian J, Xi C, Jundan H (2023). Associations of Sarcopenia, Sarcopenia parameters and motoric cognitive risk syndrome in Chinese older adults. Front Aging Neurosci.

[39] Hatabe Y, Shibata M, Ohara T (2020). Decline in handgrip strength from midlife to late-life is Associated with Dementia in a Japanese Community: the Hisayama Study. J Epidemiol.

[40] McGrath R, Vincent BM, Hackney KJ (2020). The Longitudinal associations of Handgrip Strength and cognitive function in Aging americans. J Am Med Dir Assoc.

[41] Michelle B, Priyanka D, Heather D (2018). Physical activity interventions in preventing Cognitive decline and Alzheimer-Type Dementia: a systematic review. Ann Intern Med.

[42] Xiao H, Fangfang H, Qiong W (2023). The value of Handgrip Strength and Self-rated squat ability in Predicting mild cognitive impairment: development and validation of a prediction model. Inquiry.

[43] Whitney S, Wrisley D, Marchetti G (2005). Clinical measurement of sit-to-stand performance in people with balance disorders: validity of data for the five-Times-Sit-to-stand test. Phys Ther.

[44] Haixu L, Ya F (2023). Chronic Pain increases the risk of motoric cognitive risk syndrome at 4 years of Follow-Up: Evidence from China Health and Retirement Longitudinal Study. Eur J Neurol.

[45] Yang S, Chang MC (2019). Chronic Pain: structural and functional changes in Brain structures and Associated negative Affective States. Int J Mol Sci.

[46] Wenhui Z, Lei Z, Xiangyu C (2023). Elevated dementia risk, cognitive decline, and hippocampal atrophy in multisite chronic pain. Proc Natl Acad Sci USA.

[47] Lee D, Lee K, Cho KIK (2015). Brain alterations and neurocognitive dysfunction in patients with Complex Regional Pain Syndrome. J Pain.

[48] Kim GM, Kim BK, Kim DR (2021). An association between lower extremity function and cognitive Frailty: a Sample Population from the KFACS study. Int J Environ Res Public Health.

[49] Rudd KDD, Lawler K, Callisaya MLL (2023). Investigating the associations between upper limb motor function and cognitive impairment: a scoping review. GeroScience.

[50] Darweesh SKL, Wolters FJ, Hofman A (2017). Simple test of Manual Dexterity can help to identify persons at high risk for neurodegenerative diseases in the community. The journals of gerontology. Ser Biol Sci Med Sci.

[51] Rosano C, Studenski SA, Aizenstein HJ (2012). Slower gait, slower information processing and smaller prefrontal area in older adults. Age Ageing.

[52] Cédric A, Olivier B, Sébastien C (2012). Contribution of brain imaging to the understanding of gait disorders in Alzheimer’s disease: a systematic review. Am J Alzheimer’s Dis Other Dement.

[53] Wolters FJ, Zonneveld HI, Hofman A (2017). Cerebral perfusion and the risk of dementia: a Population-based study. Circulation.

[54] McLeod KJ, Stromhaug A (2017). Reversal of cognitive impairment in a hypotensive elderly population using a passive exercise intervention. Clin Interv Aging.

[55] Wilson RS, de Leon CFM, Barnes LL (2002). Participation in cognitively stimulating activities and risk of Incident Alzheimer Disease. J Am Med Association.

[56] Lindenberger U, Baltes P (1994). Sensory functioning and intelligence in old age: a strong connection. Psychol Aging.

[57] Fischer ME, Cruickshanks KJ, Schubert CR (2016). Age-related sensory impairments and risk of cognitive impairment. J Am Geriatr Soc.

[58] Lin H, Zhang L, Lin D (2018). Visual restoration after cataract surgery promotes functional and structural brain recovery. EBioMedicine.

