# Supplementary material for: Development and validation of a risk prediction model for motoric cognitive risk syndrome in older adults
Source: Aging Clin Exp Res. 2024 Jul 13;36(1):143. doi: 10.1007/s40520-024-02797-5 (PMC11246282; doi:10.1007/s40520-024-02797-5)
Supplement: Supplementary file 1 — Supplementary Material 1 [file 40520_2024_2797_MOESM1_ESM.docx]

**Article title：**Development and validation of a risk prediction model for motoric cognitive risk syndrome in older adults

**Journal name：**Aging Clinical and Experimental Research

**Aging Clinical and Experimental Research：**

Ya-Qin Li^1^,Yu-Ting Huang^1^ ,Fang-xin Wei^1^,Tan-Jian Li^1^ and Yu Wang^2^*

1 School of Nursing, Jinan University, Guangzhou, Guangdong Province China; 2 The Community Service Center of Jinan University, The First Affiliated Hospital of Jinan University, Tianhe District, Guangzhou, Guangzhou Province, China

**Correspondence**

Yu Wang, The Community Service Center of Jinan University, The First Affiliated Hospital of Jinan University, Tianhe District, Guangzhou, Guangzhou Province, China.

Email: [hulibuwangyu@126.com](mailto:hulibuwangyu@126.com)

Table 1 Questionnaire Items of predictors

| Predictors | Questionnaire Items and definitions |
| --- | --- |
| **Social participation** |  |
| Social entertainment type | Interacted with friends; Played Ma-jong, played chess, played cards, or went to community club; Went to a sport, social, or other kind of club; Took part in a community-related organization; Attended an educational or training course; Used the Internet; Other |
| Voluntary public welfare type | Provided help to family, friends, or neighbors who do not live with you; Done voluntary or charity work; Cared for a sick or disabled adult who does not live with you |
| Economic activity type | Stock investment; Did you work for other famers in wage for at least ten days in the past year? (Agricultural work in wages); Besides agricultural work, did you work for at least one hour last week in wage or self-employed work or umpaid family business? |
| Labor participation type | Did you spend any time taking care of your grandchildren last year?; Did you work for your own household for at least ten days in the past year？ |
| **Adverse childhood experiences (ACEs)** |  |
| Threat-related ACEs |  |
| Physical abuse | When you were growing up, did your female/male guardian ever hit you?  (often, sometimes, rarely, or never) |
| Household substance  abuse | During the years you were growing up, did your female/male guardian  ever have alcoholism or drug? (yesa or no) |
| Domestic violence | Have your father/mother ever beat up your mother/father? (often, sometimes, not very often, or never) |
| Unsafe neighborhood | Was it safe being out alone at night in the neighborhood where you lived as a child? (very safe, somewhat safe, not very safe, or not safe at all) |
| Bullying | 1) When you were a child, how often were you picked on or bullied by kids  in your neighborhood? (often, sometimes, not very often, or never); 2) When you were a child, how often were you picked on or bullied by kids in your school? (often, sometimes, not very often, or never) |
| Deprivation-related ACEs |  |
| Emotional neglect | 1) How much love and affection did your female guardian give you while you were growing up? (often, sometimes, rarely, or never); 2) How much effort did your female guardian put into watching over you? (a lot, some, a little, or not at all) |
| Household mental  illness | 1) Did your female/male guardian have abnormality of mind when you  were young? (yes or no); 2) During the years you were growing up, had your female/male guardian often showed continued signs of sadness or depression? (during all, most, some, or only a little of the childhood) |
| Incarcerated  household member | During the years you were growing up, have your female/male guardian  ever been arrested or sent to prison? (yes or no) |
| Parental separation or  divorce | Were your biological parents divorced (including long separation due to  emotional problems) before you were 17 years? (yes or no) |
| Parental death | Either of the parents was dead before participant was 17 years. (yesa or  no) |
| **Childhood social isolation** |  |
| Social exclusion | 1) When you were a child, did you feel lonely because you had no friends?; 2) When you were a child, did you have a good group of friends to play with? (reverse-coded) |
| Social withdrawal | 1) Were you bullied by the neighborhood kids when you were a kid?; 2) Were you bullied at school when you were a child? |
| **ADL damaged** | Because of health and memory problems, do you have any difficulty with dressing (bathing; eating; getting in or out of bed; using the toilet; controlling urination and defecation; performing household chores; cooking; shopping; managing money; taking medication)? (do not have any difficulty, have difficulty but can still do it, have difficulty and need help or can not do it) |
| **Limb dysfunction** **score** | Do you have any difficulty with running or jogging about 1 Km (walk 1 kilometer; walk 100 meters; getting up from a chair after sitting for a long period; climbing several flights of stairs without resting; stooping, kneeling, or crouching; reaching or extending arms above shoulder level; carrying weights over 10 jin; picking up a small coin from a table)? (do not have any difficulty, have difficulty but can still do it, have difficulty and need help or can not do it) |
| **Visual acuity score** |  |
| Near visual acuity | How good is your eyesight for seeing things at a distance, like recognizing a friend  from across the street (with glasses or corrective lenses if you wear them)? (excellent, very good, good, fair, or poor) |
| Far visual acuity | How good is your eyesight for seeing things up close, like reading ordinary newspaper print (with glasses or corrective lenses if you wear them)? (excellent, very good, good, fair, or poor) |

Table 3 Comparison between variables in the training and validation datasets

| Variables | Total | Training set | Validation set | P |
| --- | --- | --- | --- | --- |
|  | 3962 | n = 2773 | n = 1189 |  |
| AMS | 16.09 (4.25) | 16.11 (4.21) | 16.04 (4.33) | 0.619 |
| Grip strength (kg) | 25.12 (8.54) | 25.11 (8.45) | 25.15 (8.76) | 0.884 |
| Limb dysfunction score | 14.10 (5.20) | 13.97 (5.14) | 14.41 (5.33) | 0.015 |
| Visual acuity score | 2.82 (0.82) | 2.81 (0.83) | 2.85 (0.80) | 0.249 |
| Childhood social isolation | 1.10 (1.33) | 1.10 (1.34) | 1.12 (1.32) | 0.635 |
| BMI | 23.14 (4.11) | 23.19 (4.14) | 23.01 (4.04) | 0.199 |
| Waist | 84.55 (13.84) | 84.73 (13.91) | 84.11 (13.67) | 0.197 |
| FTSS | 10.87 (13.84) | 10.82 (4.36) | 10.99 (4.74) | 0.286 |
| Bl-hdl | 51.72 (12.41) | 51.76 (12.48) | 51.64 (12.25) | 0.772 |
| Bl-cysc | 0.95 (0.26) | 0.95 (0.25) | 0.96 (0.28) | 0.631 |
| Bl-crp | 3.11 (6.79) | 3.14 (7.21) | 3.05 (5.69) | 0.685 |
| Bl-glu | 104.10 (32.52) | 104.67 (33.50) | 102.79 (30.09) | 0.097 |
| Bl-cho | 184.11 (35.82) | 185.03 (36.14) | 181.96 (34.99) | 0.013 |
| Bl-bun | 16.48 (4.93) | 16.45 (4.94) | 16.56 (4.91) | 0.533 |
| Bl-ua | 5.07 (1.43) | 5.08 (1.40) | 5.07 (1.51) | 0.918 |
| Bl-wbc | 5.97 (1.81) | 6.00 (1.79) | 5.91 (1.85) | 0.188 |
| Bl-hgb | 13.44 (1.84) | 13.46 (1.84) | 13.40 (1.82) | 0.384 |
| Bl-hct | 40.91 (5.59) | 40.98 (5.52) | 40.77 (5.74) | 0.274 |
| Bl-crea | 0.85 (0.31) | 0.85(0.31) | 0.86 (0.31) | 0.247 |
| Bl-ldl | 103.51 (28.84) | 104.39 (29.14) | 101.47 (28.04) | 0.004 |
| Bl-hbalc | 6.08 (0.98) | 6.09 (0.99) | 6.03 (0.96) | 0.061 |
| Systolic pressure | 134.21 (21.13) | 134.62 (21.02) | 133.27 (21.36) | 0.066 |
| Diastolic pressure | 73.98 (10.97) | 74.18 (10.97) | 73.50 (10.96) | 0.074 |
| Arthritis or rheumatism(%) | 1569 (39.6) | 1100 (39.7) | 469 (39.4) | 0.895 |
| Hypertension(%) | 1197 (30.2) | 838 (30.2) | 359 (30.2) | 0.987 |
| Malnutrition(%) | 1390 (35.1) | 949 (34.2) | 441 (37.1) | 0.083 |
| Gender (%) |  |  |  | 0.421 |
| Male | 1964 (49.6) | 1363 (49.2) | 601 (50.5) |  |
| Female | 1998 (50.4) | 1410 (50.8) | 588 (49.5) |  |
| Weakness (%) | 1421 (35.9) | 995 (35.9) | 426 (35.8) | 0.974 |
| Age, years (%) |  |  |  | 0.478 |
| ＜75 | 1750 (44.2) | 1235 (44.5) | 515 (43.3) |  |
| ≥ 75 | 2212 (55.8) | 1538 (55.5) | 674 (56.7) |  |
| Marital status(%) |  |  |  | 0.377 |
| Married | 2952 (74.5) | 2055 (74.1) | 897 (75.4) |  |
| Unmarried | 1010 (25.5) | 718 (25.9) | 292 (24.6) |  |
| Educational level(%) |  |  |  | 0.637 |
| Primary | 3257 (82.2) | 2285 (82.4) | 972 (81.7) |  |
| Secondary | 636 (16.1) | 439 (15.8) | 197 (16.6) |  |
| Tertiary | 69 (1.7) | 49 (1.8) | 20 (1.7) |  |
| Permanent address (%) |  |  |  | 0.230 |
| Urban | 993 (25.1) | 710 (25.6) | 283 (23.8) |  |
| Rural | 2969 (74.9) | 2063 (74.4) | 906 (76.2) |  |
| Agricultural work experience(%) |  |  |  | 0.985 |
| No | 1452 (36.6) | 1016 (36.6) | 436 (36.7) |  |
| Yes | 2510 (63.4) | 1757 (63.4) | 753 (63.3) |  |
| Currently smoking (%) | 1057 (26.7) | 747 (26.9) | 310 (26.1) | 0.572 |
| Currently drinking (%) | 1259 (31.8) | 885 (31.9) | 374 (31.5) | 0.776 |
| Nighttime sleep duration(%) |  |  |  | 0.081 |
| ＜6h | 1431 (36.1) | 979 (35.3) | 452 (38) |  |
| 6-8h | 2072 (52.3) | 1463 (52.8) | 609 (51.2) |  |
| ≥9h | 459 (11.6) | 331 (11.9) | 128 (10.8) |  |
| Afternoon nap(%) |  |  |  | 0.701 |
| 0h | 1595 (40.2) | 1115 (40.2) | 480 (40.4) |  |
| ＜1h | 648 (16.4) | 466 (16.8) | 182 (15.3) |  |
| ≥1h | 1719 (43.4) | 1192 (43) | 527 (44.3) |  |
| Sleep quality (%) |  |  |  | 0.905 |
| Rarely or none of the time | 2031 (51.3) | 1424 (51.4) | 607 (51.1) |  |
| Some or a little of the time | 548 (13.8) | 378 (13.6) | 170 (14.3) |  |
| Occasionally or a moderate amount of the time | 513 (12.9) | 365 (13.2) | 148 (12.4) |  |
| Most or all of the time | 870 (22) | 606 (21.9) | 264 (22.2) |  |
| Multiple morbidity (%) |  |  |  | 0.693 |
| No | 2127 (53.7) | 1483 (53.5) | 644 (54.2) |  |
| Yes | 1835 (46.3) | 1290 (46.5) | 545 (45.8) |  |
| Chonic pain (%) | 1257 (31.7) | 876 (31.6) | 381 (32) | 0.779 |
| Fall (%) |  |  |  | 0.340 |
| No | 3205 (80.9) | 2254 (81.3) | 951 (80) |  |
| Yes | 757 (19.1) | 519 (18.7) | 238 (20) |  |
| ADL damaged (%) |  |  |  | \| 0.015 \| \| --- \| |
| No | 3172 (80.1) | 2248 (81.1) | 924 (77.7) |  |
| Yes | 790 (19.9) | 525 (18.9) | 265 (22.3) |  |
| Self-perceived health status (%) |  |  |  | 0.017 |
| Good | 2833 (71.5) | 2014 (72.6) | 819 (68.9) |  |
| Poor | 1129 (28.5) | 759 (27.4) | 370 (31.1) |  |
| Hearing (%) |  |  |  | 0.083 |
| Good | 1084 (27.4) | 781 (28.2) | 303 (25.5) |  |
| Poor | 2878 (72.6) | 1992 (71.8) | 886 (74.5) |  |
| Dpression (%) |  |  |  | 0.449 |
| No depressive symptoms | 2817 (71.1) | 1981 (71.4) | 836 (70.3) |  |
| Mild depressive symptoms | 873 (22) | 606 (21.9) | 267 (22.5) |  |
| Severe depressive symptoms | 272 (6.9) | 186 (6.7) | 86 (7.2) |  |
| Aloneness (%) |  |  |  | 0.836 |
| No | 2848 (71.9) | 1996 (72) | 852 (71.7) |  |
| Yes | 1114 (28.1) | 777 (28) | 337 (28.3) |  |
| Social participation (%) |  |  |  | 0.765 |
| None | 800 (20.2) | 549 (19.8) | 251 (21.1) |  |
| Poor | 1746 (44.1) | 1247 (45) | 499 (42) |  |
| Fair | 1072 (27.1) | 741 (26.7) | 331 (27.8) |  |
| Good | 344 (8.7) | 236 (8.5) | 108 (9.1) |  |
| Hospitalization history (%) | 699 (17.6) | 469 (16.9) | 230 (19.3) | 0.066 |
| Endowment insurance (%) |  |  |  | 0.437 |
| No | 1149 (29) | 794 (28.6) | 355 (29.9) |  |
| Yes | 2813 (71) | 1979 (71.4) | 834 (70.1) |  |
| Medical insurance (%) |  |  |  | 0.713 |
| No | 414 (10.4) | 293 (10.6) | 121 (10.2) |  |
| Yes | 3548 (89.6) | 2480 (89.4) | 1068 (89.8) |  |
| Adverse childhood experience (%) |  |  |  | 0.036 |
| 0 | 82 (2.1) | 55 (2) | 27 (2.3) |  |
| 1 | 698 (17.6) | 516 (18.6) | 182 (15.3) |  |
| ≥2 | 3182 (80.3) | 2202 (79.4) | 980 (82.4) |  |

AMS,appendicular skeletal muscle mass; BMI,Body Mass Index; FTSS,Five-Times-Sit-To-Stand; Bl-hdl,high density lipoprotein cholesterol; Bl-cysc,cystatin c; Bl-crp,c-reactive protein; Bl-glu,glucose; Bl-cho,total cholesterol; Bl-bun,blood urea nitrogen; Bl-ua,uric acid; Bl-wbc,white blood cell; Bl-hgb,hemoglobin; Bl-hct,hematocrit; Bl-crea,creatinine; Bl-ldl,low density lipoprotein cholesterol; Bl-hbalc,glycated hemoglobin.
